# Can Ethical Leadership Improve Employees’ Well-Being at Work? Another Side of Ethical Leadership Based on Organizational Citizenship Anxiety

**DOI:** 10.3389/fpsyg.2020.01478

**Published:** 2020-07-28

**Authors:** Jingtao Fu, Yijing Long, Qi He, Yazhen Liu

**Affiliations:** ^1^School of Management, Hainan University, Haikou, China; ^2^Institute of Corporation Governance Research of Hainan Province, Haikou, China; ^3^School of Finance & Economics, Jiangsu University, Zhenjiang, China

**Keywords:** ethical leadership, well-being at work, organizational citizenship anxiety, organizational concern motivation, another side

## Abstract

Most of the previous literature has focused on the positive effects of ethical leadership on organizations and employees, but some studies have unexpectedly found that ethical leadership is negatively related to employees’ well-being at work. Based on the theory of workplace anxiety, this research explored whether ethical leadership can reduce employees’ well-being at work by causing them to feel anxious about organizational citizenship behavior and whether organizational concern motivation moderates this mechanism. We collected 227 three-stage time-crossed data samples from 12 institutions in Hainan China, then tested our research hypotheses to confirm that ethical leadership has a negative impact on employees’ well-being at work under certain conditions. We found that: (1) ethical leadership is significantly and positively correlated with the organizational citizenship anxiety perceived by employees, (2) organizational citizenship anxiety perceived by employees plays a completely mediating role between ethical leadership and employee well-being at work, and (3) organizational concern motivation not only negatively moderates the negative correlation between employees’ organizational citizenship anxiety and well-being at work, but also further moderates the indirect negative effect of ethical leadership on employee well-being at work through organizational citizenship anxiety.

## Introduction

People have typically associated ethical leadership with personal characteristics such as honesty, trustworthiness, fairness, principledness, and altruistic motivation (Trevino et al., 2003), and regard it as a leadership style closely related to employees’ positive organizational behaviors and attitudes (Brown and Trevino, 2006). Brown and Trevino (2006) believe that social learning theory (Bandura, 1977) and social exchange theory (Homans, 1958) can explain the personal characteristics of ethical leadership and its positive impact on employees in an organizational context. They found that ethical leadership has managerial authority, can motivate employees to pay attention, can provide a trusted and pertinent role model associated with the traits of caring and treating others fairly, and finally, provide values, attitudes, and behaviors that employees can learn and imitate. The meta-analysis conducted by Bedi et al. (2016) showed that employees working under ethical leadership demonstrate positive behaviors, willingness toward organizational citizenship, and achieving higher work satisfaction.

In contrast, some studies have identified negative effects of ethical leadership, indicating another side to the effects of ethical leadership. Yang (2014) unexpectedly found that research results are not positive when studying the relationship between ethical leadership and employees’ well-being at work. That is, ethical leadership is negatively related to employees’ well-being at work, indicating that ethical leadership may also have negative effects on employees’ organizational behaviors. Well-being is a positive and affirming experience that people strive for Waterman et al. (2008), and acknowledgment of this has become an important social consensus (Cao et al., 2019). Research shows that well-being at work is beneficial in improving employees’ work performance (Wright and Cropanzano, 2000), promoting physical and mental health (Wright et al., 2009), and reducing turnover intentions (Bonett, 1992). Therefore, the importance of well-being at work is self-evident. At present, most studies simply consider ethical leadership to be a driving force for employees’ positive organizational behaviors and attitudes in the workplace. However, the reality seems more complicated than the conclusions of existing research. We need to further explore the additional impacts of ethical leadership, taking into account employees’ well-being at work, and studying the potential mediating and moderating mechanisms that may underlie negative effects.

Based on the theory of workplace anxiety (hereinafter referred to as TWA), we considered the role of organizational citizenship anxiety when exploring the mechanisms through which ethical leadership has a negative impact on employees’ well-being at work. State anxiety refers to the state in which employees feel upset and nervous when facing work tasks that need to be completed (Eysenck et al., 2007). When employees are anxious about the organizational citizenship behavior that they need to engage in, this is referred to as organizational citizenship anxiety. Cheng and McCarthy (2018) constructed and verified TWA, proposing that organizational context is the core prerequisite for state anxiety. As an important organizational context variable, leadership style has an important impact on employees’ work attitudes and behaviors, and because leaders directly arrange employees’ work tasks, they have a greater psychological impact on employees than other members of the organization. Burke (2010) pointed out that leadership style is an important factor affecting employees’ work stress. Ethical leadership is an important leadership style. Employees engaged in organizational citizenship behaviors in the context of working under ethical leadership are more likely to generate psychological burdens. From the perspective of social learning, employees would tend to imitate ethical leadership. Zhu et al. (2016) found that ethical leadership can affect the attention employees pay to ethical issues, such that employees who work under ethical leadership are more likely to notice and internalize the ethical values and behaviors conveyed by that leadership, and make greater efforts to make their own behavior more ethical. Therefore, the decrease in employee well-being when working under ethical leadership may due to the pressure exerted by the leader on employees (Yang, 2014).

It is important to note that due to the high demand for organizational citizenship behaviors is consistent with ethical leadership, this becomes a specific work requirement for employees, and thus, individuals feel organizational citizenship pressure (Bolino et al., 2010), through facing responsibility and obligations to complete organizational citizenship behaviors. This increases state anxiety in employees. Based on the TWA, ethical leadership is associated with higher management requirements and standards for employees, and thus, employees face higher role expectations. In order to fulfill the high requirements presented by the leadership, ethical pressure and emotional anxiety are generated. We therefore proposed that ethical leadership leads to organizational citizenship anxiety in employees. The follow-up effect of this state anxiety is primarily emotional exhaustion (Smith and Ellsworth, 1985). Work tasks involving high work requirements in organizational contexts are known to evoke psychological stress in individuals and employees can perceive higher levels of potential threat, which can cause state anxiety and reduce well-being at work (Warr, 1990a). Therefore, we propose that ethical leadership motivates employees to engage in organizational citizenship behavior, which pressurizes them as well as increasing their workload and challenges. In other words, in the organizational context of ethical leadership, organizational citizenship behavior becomes a source of stress, causing individuals to experience organizational citizenship anxiety, which in turn, reduces well-being at work.

Theory of workplace anxiety proposed that the extent to which employees’ state anxiety affects their subsequent negative emotions depends on their motivation. It is known that highly motivated employees can experience more positive emotions at work. Rioux and Penner (2001) explored the important impact of organizational citizenship motivation on employee engagement in organizational citizenship behavior. Organizational citizenship motivation consists of three types of motivation: organizational concern motivation, impression management motivation, and prosocial motivation. Among these, organizational concern motivation describes the desire of employees to help the organization and integrate into it. Individuals motivated by organizational concern motivation have a positive emotional response to engage in organizational citizenship behavior (Halbesleben et al., 2010). They may also receive more positive feedback from the leaders due to their own organizational citizenship behavior and form higher-quality leadership-member exchange relationships (Lemoine et al., 2015). Therefore, the degree to which employees’ organizational citizenship anxiety can reduce well-being at work may depend on the employees’ organizational concern motivation relating to their engagement in organizational citizenship behavior. As individuals will have different responses to organizational citizenship anxiety, this study explored the moderating effect of organizational concern motivation on the negative impact of ethical leadership on employee well-being at work through organizational citizenship anxiety. It is proposed that organizational concern motivation can help to alleviate this negative impact.

## Literature Review and Research Hypotheses

### Another Side of Ethical Leadership

Brown and Trevino (2006) defined ethical leadership as an ethical behavior in personal behaviors and relationships, and the promotion of these behaviors to subordinates through two-way communication, reinforcement, and decision-making. Ethical leadership is considered to be part of effective leadership and can drive employees to participate in more positive organizational behaviors. Ethical leadership sets up a reward and punishment mechanism to urge employees to engage in ethical behavior and embeds ethics into the evaluation system of employees (Zhou et al., 2020). Brown and Trevino (2006) further explained the mechanism of ethical leadership in promoting employees to engage in organizational citizenship behavior. As ethical leadership is an attractive role model, the attention of employees becomes focused on ethical standards and normative behaviors, such that they identify and imitate ethical leadership behavior. Having received care, fairness, trust, and other related work support based on ethical leadership, employees are then prone to feel personal obligations, gratitude, the need to be trusted, and tend to work beyond the scope of their work responsibilities. Researchers have also found that employees who work under ethical leadership tend to perceive the importance of their work tasks in achieving organizational goals and their own value, and they also have greater autonomy (Piccolo et al., 2010). Therefore, most studies have focused on the positive effects of ethical leadership on employees’ performance (Walumbwa et al., 2011; Zhu et al., 2015; Byun et al., 2018), with findings supporting the conclusion that ethical leadership will result only in positive effects. Thus, ethical leadership is generally believed to always bring positive effects to the organization and employees, and not have any negative impacts.

However, Stouten et al. (2013) have argued that in practice, ethical leadership can have the opposite effect on employees’ positive organizational behavior. They proposed that there is an inverse u-shaped relationship between ethical leadership and employees’ organizational citizenship behaviors. This is because working under ethical leadership, employees are more likely to consider their own ethical level to be dwarfed, worry that ethical leaders will assess whether the values behind their attitudes and behaviors are in compliance with the norms, and thus may question them. Due to the different levels of ethical cognition of both parties, employees tend to think that ethical leaders would consider them ethically lacking, then afraid of moral reproach. Moreover, employees work under ethical leadership have to maintain high levels of organizational citizenship behaviors. If employees’ organizational citizenship behaviors are reduced slightly and cannot meet the expectations of leaders, they will feel anxious. This perspective demonstrates that positive effects may not be the only outcome of ethical leadership. Therefore, we need to explore the negative effects that ethical leadership may bring to better understand how the effectiveness of ethical leadership can be increased.

### Ethical Leadership and Organizational Citizenship Anxiety

Organizational citizenship behavior is considered to be a discretionary behavior conforming to the ethics of an organization. It can promote the effective operation of the organization, including the willingness to help colleagues complete work tasks and actively participate in various affairs in the organization (Organ, 1988). When considering the role of ethical leadership in promoting organizational citizenship behavior, it can be argued that employees working under ethical leadership attach great importance to organizational citizenship behavior. Research by Deery et al. (2016) found that the increased initiative of individuals to engage in organizational citizenship behavior is accompanied by increased work stress and role overload. Thus, employees who work under ethical leadership will face more work pressure and role overload when engaging in organizational citizenship behavior, which means that employees can perceive greater time pressure, state anxiety, and worry about work requirements related to organizational citizenship behavior (Thatcher, 2007). State anxiety refers to a state of pain or physiological arousal when responding to stimuli including new situations and potential adverse outcomes (Brooks and Schweitzer, 2011). Spielberger (1985) defined state anxiety as the tendency to experience tension and worry in the assessment of threatening situations. Therefore, organizational citizenship anxiety can be defined as a specific type of work state anxiety, that is, the emotional state of tension and nervousness of an employee when facing the work demand of organizational citizenship behavior.

Theory of workplace anxiety stated that an individual’s organizational context can generate a variety of stresses that affect his or her state anxiety level, and the strain of an individual’s stress in the organizational context determines subsequent state anxiety level (Cheng and McCarthy, 2018). When ethical leadership encourages individuals to engage in organizational citizenship behavior, it may cause employees to have organizational citizenship anxiety from two aspects. Ethical leadership can strengthen the ethical behaviors of employees by exerting role models and setting reward and punishment mechanisms, establishing high-quality leader-member exchange relationships with employees, providing employees with support, care, trust, and resources, and drive employees to reward positive attitudes and behaviors that leaders value based on the principle of reciprocity. Therefore, employees who work under ethical leadership face higher expectations in terms of work engagement and organizational citizenship behavior (Bedi et al., 2016). However, employees’ active participation in organizational citizenship behavior is accompanied by overloaded workload (Deery et al., 2016) and more complex work arrangements (Somech, 2015), such as whether they can complete the tasks related to organizational citizenship behavior on time and in sufficient quantity. This makes employees believe that completing organizational citizenship behaviors according to leaders’ expectations poses a potential threat to them because of the inherent uncertainty (Rodell and Judge, 2009). Consistent with Warr’s (1990b) study, work state anxiety is significantly and positively related to work overload and uncertainty. State anxiety is a stress response to uncertainty, expressing the expected emotional response to a specific or potential threat to an event (Muschalla, 2017). Therefore, employees are likely to have state anxiety perception when engaging in organizational citizenship behavior under the context of ethical leadership.

Some scholars have found that employee performance related to organizational citizenship behaviors is closely related to organizational efficiency and profitability. Accordingly, leaders should not only consider their in-role behaviors but also their organizational citizenship behaviors when evaluating employees’ performance (Podsakoff et al., 2009). Rotundo and Sackett (2002) found that performance related to organizational citizenship behaviors accounted for a large proportion of the overall performance appraisal of employees. In this case, organizational citizenship anxiety arises when employees face tasks that require them to complete organizational citizenship behaviors. Therefore, when employees engage in organizational citizenship behaviors in the context of ethical leadership, they need to pay more attention to the work requirements related to organizational citizenship behavior, such as the interests of the organization, colleagues, and customers, to improve work performance and meet the expectations of the ethical leader. When employees engage in organizational citizenship behavior under pressure, organizational citizenship behavior is no longer a discretionary behavior and they will feel restless when they anticipate that they cannot achieve the corresponding results and meet the expectations of ethical leaders (Yam et al., 2017).

### Employees’ Organizational Citizenship Anxiety and Well-Being at Work

Well-being at work is one dimension of employee well-being. Zheng et al. (2015) defined well-being at work as employees’ perceptions and feelings of work satisfaction, their psychological experiences and satisfaction level at work, and work-related positive and negative emotions. According to TWA, state anxiety can lead to emotional exhaustion and reduced work performance. Elst et al. (2014) proposed that employee emotional exhaustion in the workplace is an indicator of well-being at work, which is manifested by the exhaustion of an individual’s feelings for a certain thing (Chang, 2009). As a result, employees have negative experiences and negative work emotions, such as impaired self-esteem, depression, nervousness, and irritability (Guillaume et al., 2018). In addition, employees with state anxiety tend to exhaust their energy and attention to worry about task-related issues and criticize their abilities (Sarason, 1984). When such worry exceeds a certain level, it will hinder employees’ work satisfaction (Boyd et al., 2009) and work motivation, so it is difficult for employees to improve their work performance (Wu et al., 2020).

Organizational citizenship anxiety reflects employees’ nervous mentality about engaging in organizational citizenship behaviors. Employees with organizational citizenship anxiety tend to worry that engaging in organizational citizenship behaviors will not achieve the expected results, thereby damage their work satisfaction and motivation, then affect their performance evaluation by the leader on task-related organizational citizenship behaviors. When employees are unable to meet the expectations of leaders and the realization of their own values, the specific and potential threats generated in the process of engaging in organizational citizenship behaviors will create insecurity which makes employees feel irritable and pained, reducing their well-being at work (Elst et al., 2014). Warr’s (1990b) research took state anxiety as a predictor of employees’ well-being, and it was found that state anxiety is closely related to decreased well-being at work. When work state anxiety leads to a decrease in work performance, it means that employees feel frustrated with their work achievement. Employees’ well-being at work is related to the satisfaction in work achievement (Zheng et al., 2015), and when the satisfaction in work achievement is reduced, their well-being may also be reduced.

### The Mediating Effect of Employee Organizational Citizenship Anxiety

Subordinates can feel leaders’ higher expectations and requirements for ethical behaviors when they work under ethical leadership. Ethical leaders expect employees to engage in altruistic motivated organizational citizenship behaviors, such as helping colleagues, organizations, or other stakeholders. TWA proposed that the promoting effect of ethical leadership in the organizational context of employees’ engaging in organizational citizenship behavior will be considered as a threatening task requirement by employees, which will cause state anxiety about engaging in organizational citizenship behavior. Inducing organizational citizenship anxiety in this way can lead to emotional exhaustion of employees (Cheng and McCarthy, 2018), causing cognitive interference and distraction, as well as negatively affecting the psychological and physical health of employees at work.

Ethical leaders are ethical managers, using reward and punishment mechanisms to urge their employees to engage in ethical behaviors such as organizational citizenship behaviors, and they tend to include employee performance relating to organizational citizenship behavior into the scope of performance evaluation (Rotundo and Sackett, 2002). Thus, an ethical leader is an ethical individual, who cares about employees and treats them with integrity and fairness. Employees often trust and appreciate the ethical leader, especially when they get more care, and fair and trusted treatment through communication and exchange. Furthermore, employees tend to think that they are in a high-quality social exchange relationship with their leaders, so that they should engage in organizational citizenship behaviors to reward their experiences of being actively treated in the work process (Trevino and Brown, 2000). These two factors mean that in the organization where ethical leadership is provided, employees engaging in organizational citizenship behavior do so not only because it is necessary but also to achieve results. They need to be able to meet the expectations of leaders to improve the results of their performance evaluation. This can cause employees to feel uneasy and nervous about the expected effects of organizational citizenship behaviors. When the influence of ethical leadership and the quality of leaders’ social exchange relationships with employees are high, employees are more likely to perceive that engaging in organizational citizenship behaviors is a work requirement, causing them to face more pressure regarding organizational citizenship behaviors, resulting in organizational citizenship anxiety. As a result, employees’ positive emotional resources are consumed. Therefore, employees will feel emotional exhaustion, experience negative emotions relating to work, and reduce well-being at work. Based on the above analysis, we proposed the following hypotheses:

Hypothesis 1: Employee organizational citizenship anxiety mediates the relationship between ethical leadership and well-being at work.

### The Moderating Effect of Organizational Concern Motivation

Organizational concern motivation is one of the three dimensions of organizational citizenship behavior motivation. Employees tend to engage in organizational citizenship behaviors because organizations provide them with a satisfying work environment, fair treatment, inspiring leadership, and are supportive in providing interesting work content (Bolino and Turnley, 2003). Organizational concern motivation refers to the fact that employees perceive that the organizational situation meets their specific needs. Being concerned about the interests of the organization is an important motivation for them to engage in organizational citizenship behaviors. Highly motivated individuals value the responsibility of engaging in organizational citizenship behaviors and fulfilling civic ethics. In their view, engaging in organizational citizenship behavior is appropriate, and doing so is beneficial to the long-term development of the organization. Therefore, they are willing to engage in exceptional and time-consuming discretionary behaviors, undertake tasks that are important to the success of the organization but cannot be sufficiently rewarded (Grant, 2008). Even when face the pressures of organizational citizenship behavior brought by leaders, they will not react negatively because of feeling in control. On the contrary, they have a higher level of professional ethics and are more willing to become contributors and experts in the workplace (Klotz et al., 2017), can engage in organizational citizenship behaviors with positive emotions (Halbesleben et al., 2010), and will not experience excessive loss of psychological resources but instead of feeling satisfaction at work.

When face organizational citizenship anxiety, employees motivated by organizational concern motivation have a stronger willingness to respond and experience positive emotions. Individuals with high organizational concern motivation pay more attention to organizational interests, put organizational interests above their own goals, and are willing to take risks to complete tasks that they think are right, which can alleviate the tension and state anxiety of engaging in organizational citizenship behavior. According to TWA, organizational concern motivation can immerse employees into the completion of work tasks and lead them to be energetic, positively contribute to organizational citizenship behaviors, and realize the positive significance of engaging in organizational citizenship behaviors. In contrast, individuals with low organizational concern motivation tend to consider the adverse consequences when engaging in organizational citizenship behaviors. When employees consider challenging and time-consuming organizational citizenship behaviors as work threat and cannot face it with confidence, this excessive state anxiety will lead to emotional exhaustion and a negative approach toward organizational citizenship behavior. Employees thus tend to feel that it takes a lot of effort to achieve the results, and it is difficult to meet the expectations of leaders, but easy to adversely affect the results of their performance evaluation. This will exacerbate the loss of the resources caused by emotional deterioration, and decrease well-being at work. We therefore made the following assumption:

Hypothesis 2: Organizational concern motivation moderates the relationship between organizational citizenship anxiety and well-being at work. The higher the organizational concern motivation, the weaker the negative impact of organizational citizenship anxiety on well-being at work.

Based on the above assumptions, ethical leadership has an indirect effect on well-being at work through employees’ organizational citizenship anxiety. TWA proposed that motivation can be used as a boundary condition for workplace state anxiety. Therefore, based on organizational citizenship anxiety, this study introduces organizational concern motivation as a specific boundary condition. Individuals with high organizational concern motivation are firm in their responsibility and willingness to help the organization, and take appropriate actions based on organizational identity (Rioux and Penner, 2001). Organizational concern motivation is reflected in the ability of employees to adapt the leadership style of ethical leadership based on altruism and placing organizational interests first. Employees will actively engage in organizational citizenship behavior in accordance with the norms of ethical leadership, and be able to overcome the challenges encountered in engaging in organizational citizenship behavior, and respond to organizational citizenship anxiety with positive emotions. It can be considered that the stronger the organizational concern motivation of employees engaging in organizational citizenship behavior, the more willing they are to meet the expectations of ethical leader, the stronger their confidence and willpower to engage in organizational citizenship behavior, and the less they will worry about the potential risks of organizational citizenship behavior. Instead, they will actively engage in work, enhance their vitality in the work, and believe that their work is valuable and meaningful. On the contrary, the weaker the employees’ organizational concern motivation, the more they will tend to worry that they are unable to meet the expectations of ethical leadership relating to organizational citizenship behavior, experience higher levels of state anxiety in organizational citizenship behavior, and be more likely to experience emotional exhaustion and unable to perform core work duties. As a result, the task performance level is lowered, employees are more likely to feel stress and depression at work, and experience reduced well-being at work. Therefore, we proposed the following hypothesis:

Hypothesis 3: Organizational concern motivation negatively moderates the mediating effect of organizational citizenship anxiety between ethical leadership and well-being at work. When organization concern motivation is high, the mediating effect of organizational citizenship anxiety will be weakened.

**FIGURE 1:**
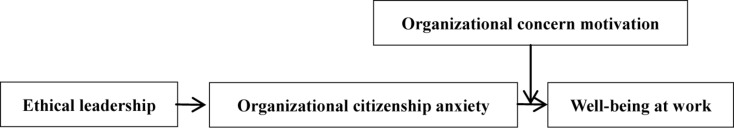
Theoretical model.

To summarize, this study aimed to explore the underlying connections between ethical leadership, organizational citizenship anxiety, and well-being at work. It further sought to verify the mechanism by which ethical leadership reduces well-being at work through enhancing organizational citizenship anxiety in employees, and explore the potential moderating effect of organizational concern motivation on the relationship between organizational citizenship anxiety and well-being at work using a moderated mediation model. The possible theoretical contributions of this study are three-fold: First, the reliability of the research results regarding the negative influence of ethical leadership on employees’ well-being at work derived from Yang (2014) was tested to further verify the impact of ethical leadership on employees’ well-being at work. Second, use TWA to test the mechanism underlying the negative effects of ethical leadership, to define the mediating role of organizational citizenship anxiety, and to deepen the understanding of the causes and effects of organizational citizenship anxiety. Third, define a boundary condition of organizational citizenship anxiety affecting employees’ well-being at work, and test the moderating effect of organizational concern motivation, which integrates the relevant research conclusions regarding organizational citizenship behavior motivation and organizational citizenship anxiety. The proposed theoretical model is shown in Figure 1.

## Research Design

### Research Samples and Procedures

The research samples consisted of employees from 12 enterprises and institutions in Hainan Province of China. As the research questions are relatively obscure, we chose organizations we had good relationships and who were cooperative about conducting the questionnaire survey. After the initial questionnaire was created, we first selected 10 sample participants to undergo a preliminary questionnaire interview, then we modified the semantic expression and other issues. After this modification, the questionnaire with the setting code was used to conduct a time-crossed formal survey. Therefore, due to the modification of the questionnaire, the content validity of the questionnaire was ensured to some extent. We first contacted the human resource managers of the organizations, and after obtaining their consent, we provided training and explanation to the employees about the meaning of each variable and how to fill the questionnaire. To ensure the authenticity of the data, the participants were informed that the survey data were anonymous and would be only used for academic research. Three survey phases were completed using the coded questionnaire. The first phase obtained demographic information, perceived ethical leadership, and organizational concern motivation. The second phase of the survey was conducted 2 months after the end of the first phase of the survey, to examine the organizational citizenship anxiety of employees after they felt the ethical leadership. The third phase of the survey was conducted 2 months after the second phase of the survey, to observe the impact of employees’ organizational citizenship anxiety on their well-being at work.

Paper questionnaires and electronic questionnaires were used. For organizations close to us, we used paper questionnaires. For organizations that are far away from us, we used electronic questionnaires. In the first phase, 320 questionnaires were distributed and 287 were returned. In the second phase, 320 were distributed and 245 were returned. In the third phase, 320 questionnaires were distributed and 253 were returned. A total of 227 valid questionnaires were successfully matched. The response rate was 70.94%. The distribution characteristics of the effective samples are shown in Table 1.

**TABLE 1:** Sample distribution characteristics.

**Variable**	**Category**	**Proportion**
Gender	Male	34.40%
	Female	65.60%
Age	≤25	8.80%
	25–30	55.10%
	30–40	34.40%
	40–50	0.90%
	50–60	0.90%
Education	Bachelor’s degree or above	98.60%
	The rest	1.40%
Working years	≤1	6.60%
	1–3	20.30%
	3–5	36.60%
	5–7	19.80%
	>7	16.70%
Years of working with the direct leader	≤1	24.20%
	1–3	41.90%
	3–5	22.50%
	5–7	7.50%
	>7	4.00%
Type of work organization	Public funding involved	78.00%
	No public funding involved	22.00%

### Measurement Tools

All the scales in this study were directly or indirectly derived from the established scales used in the literature. The English scales were translated and back-translated according to the procedure designed by Brislin (1980). All responses were measured using the Likert five-point scoring method.

#### Ethical Leadership

The 10-item scale used by Brown et al. (2005) was referenced. The Cronbach’s reliability coefficient of the scale is 0.931. Representative measurement items include “My leader’s definition of success lies not only in results, but also in the way to get results,” and so on.

#### Organizational Citizenship Anxiety

According to the state anxiety scale used by McCarthy et al. (2016), an organizational citizenship anxiety scale with a total of eight items was designed. The Cronbach’s reliability coefficient of the scale is 0.865. It measured the level of state anxiety generated by employees when they engaged in organizational citizenship behaviors. First, since we wanted to measure organizational citizenship anxiety, we excluded the unreasonable items in the original scale (for example, the original content “I worry that whether others consider me as a good employee for the work,” considering that in Chinese culture, employees’ state anxiety often comes from the leader, so their focus is mainly on the leadership, and seldom consider others’ opinions on whether they are competent or not. Therefore, this item is not suitable in China, then we deleted it) and then modified the questions (for example, the original content “I worry that my work performance will lower than that of others at work” was changed to “I worry that my organizational citizenship behavior is less than others”) to assess organizational citizenship anxiety. The factor load in exploratory factor analysis of the scale was above 0.6 and indicated one factor. Several indexes of the confirmatory factor analysis were within a reasonable range, indicating that this scale was appropriate for using in this study. Second, we explained the definition of organizational citizenship behavior to employees based on the classic literature by Organ (1988), and then listed several typical organizational citizenship behaviors in the workplace (e.g., helping absent colleagues) to help survey respondents accurately understand the concept of organizational citizenship behavior.

#### Well-Being at Work

The employee’s well-being scale developed by Zheng et al. (2015) was used, more specifically, the subscale of well-being at work was used. There are six items with a Cronbach’s reliability coefficient of 0.864. Representative items include “I am basically satisfied with my specific work content.”

#### Organizational Concern Motivation

The organizational citizenship behavior motivation scale developed by Rioux and Penner (2001) was used, and the organizational concern motivation dimension was used as the moderating variable. This scale has 10 items, and the Cronbach’s reliability coefficient of the scale is 0.832. Items include “Because I want to understand how the organization works,” and so on.

#### Control Variables

Since age and gender may have an impact on the results (Resick et al., 2013), we used employee gender and age as control variables. Since the working years and years of working with the direct leader can result in employee’s work inertia and emotional dependence (Richards and Hackett, 2012), we speculated that these would also affect the findings and used them as control variables. Moreover, employees from diverse sets of organizations differ in nature may have different expectations for organizational citizenship behaviors and differ in the requirements for academic qualifications. We speculated that these variables would affect the findings and so type of work organization and education were also used as control variables. Here, we used dummy variables to encode the type of work organization, public funding involved coded 1, and no public funding involved coded 0.

## Data Analysis and Results

### Common Method Deviation Test and Confirmatory Factor Analysis

As all the data were obtained via self-report, there may be common method deviation. Therefore, we adopted Harman single factor analysis to carry out exploratory factor analysis for all the items across the four variables. The results showed that KMO = 0.858, the Chi-square value of Bartlett’s spherical test value was 7560.199, and the *p*-value was less than 0.001. The four common factors extracted were consistent with the number of variables set in this study, and the variability of the first factor was 20.783%, lower than the critical value of 50%, so the data were deemed not to have serious common method bias issues. In addition, data were obtained anonymously, and the research procedures were strictly controlled to control the common method deviation.

AMOS 24.0 software was used to test the validity of the variables. The results of the confirmatory factor analysis are shown in Table 2. Compared with other models, the four-factor model fit the index best (χ^2^/df = 1.462; RMSEA = 0.045; RMR = 0.046; CFI = 0.930; IFI = 0.931; TLI = 0.924). This confirmed that the factors of ethical leadership, organizational citizenship anxiety, well-being at work, and organizational concern motivation have good discriminative validity.

**TABLE 2:** Confirmatory factor analysis.

**Model**	**χ^2^**	**df**	**χ^2^/df**	**RMSEA**	**RMR**	**CFI**	**IFI**	**TLI**
Four factors F	761.465	521	1.462	0.045	0.046	0.930	0.931	0.924
Three factors E	1273.088	524	2.430	0.080	0.086	0.781	0.784	0.766
Three factors D	1303.356	524	2.487	0.081	0.102	0.772	0.673	0.756
Two factors C	1893.068	526	3.599	0.107	0.138	0.601	0.605	0.574
Two factors B	1897.945	526	3.608	0.107	0.136	0.599	0.604	0.573
One factor A	2424.045	527	4.600	0.126	0.154	0.446	0.452	0.410

*(A) Ethical leadership + organizational citizenship anxiety + well-being at work + organizational concern motivation. (B) Ethical leadership + organizational citizenship anxiety + well-being at work; organizational concern motivation. (C) Ethical leadership + organizational citizenship anxiety; well-being at work + organizational concern motivation. (D) Ethical leadership; organizational citizenship anxiety; well-being at work + organizational concern motivation. (E) Ethical leadership; organizational citizenship anxiety + organizational concern motivation; well-being at work. (F) Ethical leadership; organizational citizenship anxiety; well-being at work; organizational concern motivation.*

### Descriptive Statistics and Correlation Analysis

Table 3 shows mean, standard deviation, and correlation coefficient. As can be seen from Table 3, ethical leadership is significantly and positively correlated with organizational citizenship anxiety (*r* = 0.209, *p* < 0.01). That is, ethical leadership is significantly and positively related to organizational citizenship anxiety perceived by employees. Organizational citizenship anxiety is significantly and negatively correlated with well-being at work (*r* = −0.349, *p* < 0.01). Thus, organizational citizenship anxiety perceived by employees is significantly and negatively related to employee’s well-being at work. This provided the basis for further testing the research hypotheses.

**TABLE 3:** Descriptive statistics and correlation analysis.

**Variable**	**Mean**	**SD**	**1**	**2**	**3**	**4**	**5**	**6**	**7**	**8**	**9**	**10**
(1) Gender	0.34	0.476	1									
(2) Age	2.30	0.677	0.146*	1								
(3) Education	3.85	0.666	–0.009	0.225**	1							
(4) Working years	3.20	1.141	0.070	0.255**	–0.026	1						
(5) Years of working with the direct leader	2.25	1.032	0.013	0.120	0.015	0.412**	1					
(6) Type of work organization	0.78	0.415	0.116	0.110	0.252**	0.130	0.078	1				
(7) Ethical leadership	3.58	0.740	0.044	0.020	–0.026	0.025	0.045	−0.137*	1			
(8) Organizational citizenship anxiety	2.99	0.704	–0.006	0.047	–0.105	–0.064	–0.037	−0.137*	0.209**	1		
(9) Well-being at work	3.34	0.774	–0.009	0.037	0.017	0.112	0.007	0.062	−0.136*	−0.349**	1	
(10) Organizational concern motivation	2.83	0.557	0.072	0.029	–0.078	0.025	–0.007	–0.100	0.041	–0.111	0.152*	1

*p < 0.05, **p < 0.01.

### Hypothesis Testing

#### The Mediating Effect of Organizational Citizenship Anxiety

In this study, SPSS macro Process developed by Hayes (2013) was used to further test the mediating effect. The 95% confidence interval (CI = [−0.1426, −0.0224]) of the Bootstrapping for the mediating effect of organizational citizenship anxiety excludes 0. The result indicated that the mediating effect of organizational citizenship anxiety was significant, and the effect value is −0.0699 (*SE* = 0.0307), supporting our hypothesis about mediation.

#### The Moderating Effect of Organizational Concern Motivation

**FIGURE 2:**
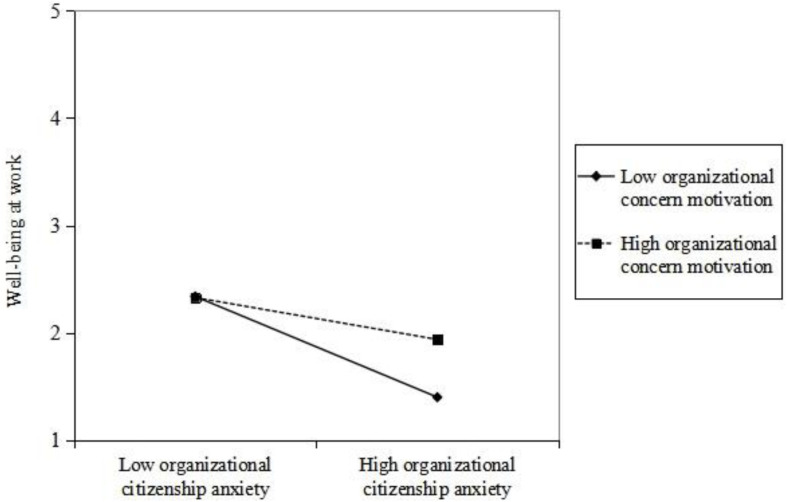
The moderating effect of organizational concern motivation.

As can be seen from model 4 in Table 4, organizational citizenship anxiety was significantly and negatively correlated with “well-being at work” (β = −0.325, *p* < 0.001), and the interaction term “organizational citizenship anxiety × organizational concern motivation” was significantly and positively correlated with “well-being at work” (β = 0.144, *p* < 0.05). The coefficients of the two are in opposite directions. The organizational concern motivation of employees plays an interfering moderating role between perceived organizational citizenship anxiety and well-being at work, supporting our hypothesis. Figure 2 was then modified to show the direction and trend of the moderating effect more intuitively. Compared with employees with high organizational concern motivation, organizational citizenship anxiety has a more significant impact on the well-being at work of employees with low organizational concern motivation.

**TABLE 4:** The moderating effect of organizational concern motivation.

**Variable**	**Well-being at work**			
	**Model 1**	**Model 2**	**Model 3**	**Model 4**
**Control variable**				
Gender	–0.024	–0.025	–0.034	–0.026
Age	0.007	0.043	0.037	0.023
Education	0.007	–0.029	–0.020	–0.014
Years of working	0.126	0.100	0.098	0.115
Years of working with the direct leader	–0.049	–0.052	–0.051	–0.059
Type of work organization	0.049	0.011	0.024	0.030
**Mediating variable**				
Organization citizenship anxiety		−0.348***	−0.332***	−0.325***
**Moderating variable**				
Organization concern motivation			0.114	0.134*
**Interaction term**				
Organization citizenship anxiety × Organization concern motivation				0.144*
*F*	0.651	4.844***	4.683***	4.815***
Δ*F*	0.651	29.495***	3.211	5.161*
*R*^2^	0.017	0.134	0.147	0.166
Δ*R*^2^	0.017	0.117	0.013	0.020

*p < 0.05, ***p < 0.001.

#### The Moderated Mediating Effect

We then used SPSS macro Process developed by Hayes (2013) to test the moderated mediating effect. The moderating variable of organization concern motivation was divided into three groups: high, medium, and low, according to the mean plus or minus one standard deviation, to compare the mediating effect of organizational citizenship anxiety between ethical leadership and well-being at work at different levels of organizational concern motivation. The results are shown in Table 5. Under the low-level organizational concern motivation, the indirect effect of ethical leadership on well-being at work was significant (95% CI = [−0.2045, −0.0288]). Under the high-level organizational concern motivation, the indirect effect value was less than the value of low-level organizational concern motivation status and still significant (95% CI = [−0.1034, −0.0019]). This shows that the lower the organizational concern motivation, the more significant the mediating effect of organizational citizenship anxiety between ethical leadership and well-being at work. Therefore, our hypothesis was supported.

**TABLE 5:** The moderated mediating effect.

**Group**	**Effect**	***SE***	**BootLLCI**	**BootULCI**
Low organizational concern motivation	−0.0972	0.0439	−0.2045	−0.0288
Medium organizational concern motivation	−0.0677	0.0301	−0.1414	−0.0199
High organizational concern motivation	−0.0382	0.0250	−0.1034	−0.0019

*N = 227. The three values of the organizational concern motivation are the mean and one standard deviation above and below. SE, standard error.*

## Research Conclusion and Discussion

This research focused on the question of why ethical leadership has an adverse effect on employee’s well-being at work. Most recent studies on ethical leadership have demonstrated that ethical leadership is an important leadership style that affects employees’ organizational citizenship behavior (Brown and Trevino, 2006; Bedi et al., 2016). Employees who work under ethical leadership will perceive supportive, trusting, and positive treatment from leaders. Accordingly, they will feel more positively about their work, such as experiencing higher work satisfaction (Bedi et al., 2016), as well as a stronger sense of responsibility and security regarding their work (Liang, 2014). However, research by Yang (2014) found that working under ethical leadership is not necessarily positive and may even be harmful to employees. Therefore, in the process of working under leaders’ leadership and building a high-quality social exchange relationship with them, the support that employees receive from the leaders also results in greater pressure. In other words, ethical leadership may have a negative impact on employees’ well-being at work.

### Research Results

In the context of Yang (2014)’s research conclusions that there may be a negative relationship between ethical leadership and employees’ well-being at work, we introduced organizational citizenship anxiety as a mediating variable based on TWA and proposed a mechanism based on the mediating effect of organizational citizenship anxiety in the relationship between ethical leadership and employees’ well-being at work. The data were collected through questionnaire surveys of employees across 12 institutions in Hainan, China. It was found that ethical leadership will lead to employees experiencing organizational citizenship anxiety, which will reduce their well-being at work. The organizational citizenship anxiety perceived by employees is a mediator on how ethical leadership negatively affects well-being at work and it plays a fully mediating role. When employees engage in organizational citizenship behaviors, their organizational concern motivation moderates the relationship between organizational citizenship anxiety and well-being at work. The higher an individual’s organizational concern motivation, the weaker the negative impact of organizational citizenship anxiety on well-being at work. In addition, organizational concern motivation also moderates the indirect negative effect of ethical leadership on employees’ well-being at work through organizational citizenship anxiety. For employees with high organizational concern motivation, the mediating role of organizational citizenship anxiety is weak.

### Theoretical Contributions

The theoretical contributions of this research are mainly reflected in three ways. First, the use of TWA has extended the existing research on the negative effects of ethical leadership and enriched the theoretical perspective of employees’ well-being at work. Previous studies on ethical leadership have mostly explored the impact of ethical leadership on employees’ positive organizational behaviors and attitudes based on social learning theory and social exchange theory. Most of these studies considered the positive impact of specific leadership styles on organizations and employees from the perspective of the leaders, as well as the work tasks undertaken by employees in the organization, including the promotion of effective organization operations, employee advice, and active behavior (Song and Xia, 2011; Walumbwa et al., 2011; Wu et al., 2017). However, few studies have explored the cost of working under ethical leadership from the perspective of employees, which reduces well-being at work. This study focused on the well-being of employees, using TWA to verify the other side of ethical leadership, which enriches the theoretical understanding of employee well-being at work.

Second, a more complete mechanism was constructed by introducing organizational citizenship anxiety. The dark side of ethical leadership has been discussed (Yang, 2014), enlightening us to further explore the negative effect of ethical leadership. This study used TWA to explain the mechanism through which ethical leadership has a negative impact on employee well-being at work, and tested the mediating effect of organizational citizenship anxiety in the relationship between ethical leadership and employee well-being at work. It confirmed that ethical leadership reduces employee well-being at work by increasing organizational citizenship anxiety, further validated the conclusions by Yang (2014), and explained why ethical leadership is negatively related to employee well-being at work. Eisenbeiss (2012) summarized four core ethical orientations of ethical leadership, two of which are responsibility and sustainability orientation, and moderation orientation. Responsibility and sustainability orientation emphasizes that leaders focus on the long-term development of the organization, and requires to strengthen individual’s sense of responsibility toward the organization. Moderation orientation emphasizes that leaders should be modest, temperate, and self-controlled, have the ability to suppress self-emotions and desires, and can sacrifice their own interests for the goals of the organization or relevant stakeholders. Research by Mao et al. (2017) showed that employees are more willing to be close to humble leaders and then engage in organizational citizenship behaviors. Although meta-analysis of Hoch et al. (2018) showed transformational leadership has a stronger predictive and explanatory power on organizational citizenship behavior than ethical leadership, they may filter out subordinates’ critical thoughts that are inconsistent with their own views and force their own vision forward (Tourish, 2013; Li and Yuan, 2017). Organizational citizenship behavior contains a strong ethical element (Yam et al., 2017), which is more closely related to ethical leadership. Therefore, organizational citizenship behavior is selected as the entry point in the study because employees that work under ethical leadership engage in organizational citizenship behaviors and cater to the value orientations of ethical leadership. In this context, introducing organizational citizenship anxiety to build a theoretical model explains the negative effects of ethical leadership, can enhance our understanding of the effects of ethical leadership.

Third, based on TWA, the moderating variable of organizational concern motivation was introduced to improve the context adaptability of the theoretical model that explains the negative influence of ethical leadership on employees’ well-being at work. Organizational concern motivation is the boundary condition of the relationship between employees’ organizational citizenship anxiety and well-being at work. In this study, the moderating role of organizational concern motivation is elaborated and verified. In the case of ethical leadership exacerbating employees’ organizational citizenship anxiety, it further explained how organizational citizenship motivation enhances or reduces employee well-being at work, thus integrated theoretical research on organizational citizenship behavior motivation and organizational citizenship anxiety.

### Practical Inspiration

First, ethical leadership is gradually becoming the most important factor affecting the positive performance of employees, including the provision of continuous investment and interpersonal help (Song and Xia, 2011). This study considered the psychological feelings related to positive performance in employees to explore whether there is another side of the leadership style favored by contemporary organizations. In the other words, we tested whether it would lead to negative effects, more specifically, whether ethical leadership encourages employees to engage in organizational citizenship behaviors that will produce state anxiety and reduce well-being at work. Our findings confirmed that ethical leadership is an organizational situation that causes organizational citizenship anxiety in employees. Therefore, managers should try to avoid arranging work tasks that may cause employee state anxiety and leading to difficult organizational citizenship behaviors.

Second, ethical leaders should recognize and deal with the negative psychological feelings that employees may have when respond to work tasks related to organizational citizenship behaviors. Previous studies have suggested that state anxiety generated in the workplace is costly, and employees with state anxiety are more likely to experience emotional failure, leading to lower levels of work performance (Ford et al., 2011). Therefore, for employees with organizational citizenship anxiety, if it is difficult to prevent them from engaging in organizational citizenship behaviors, it is necessary to provide them with more relevant resources to engage in organizational citizenship behavior and thus compensate for the loss of self-control resources resulting from state anxiety.

Finally, management should focus on supporting employees’ organizational concern motivation. Organizational concern motivation is more determined by organizational context. When employees engage in organizational citizenship behaviors based on organizational concern motivation, they will show a strong desire to help the organization and a high level of organizational commitment provided that the organization provides them with satisfying organizational support (Bolino and Turnley, 2003). If an organization wants to increase the level of organizational concern motivation of employees, it needs to provide more cognitive interventions for employees to strengthen this motivation (for example, the organization can improve the knowledge and expertise of employees through education and training, so that employees can feel the support and help from the organization, and improve their intuitive and deliberate consideration, so as to increase the level of organizational concern motivation; strengthening the communication between employees through collective activities can let employees get help from other colleagues, also can enhance the level of organizational concern motivation), also needs to pay attention to screening employees with low organizational concern motivation during recruitment.

### Research Limitations and Prospects

First, we performed hypothesis testing using the collected time-crossed data, the negative effects of ethical leadership may not be completely accurate. Follow-up studies should use more objective data to explore the impact of ethical leadership on employees’ negative psychology. Collecting leadership-employee pairing data and conducting further longitudinal research would be beneficial. The second limitation is that although we considered potential influencing factors such as organizational citizenship anxiety, organizational concern motivation, and demographic information, we did not consider any other variables that may affect the negative impact of ethical leadership on employee well-being at work, such as job insecurity (Mahlagha and Faizan, 2020), or the quality of coworker exchange and leader-member exchange (McCarthy et al., 2016). Organizational citizenship behavior, trait anxiety, negative affect, or neuroticism, may also influence the relationship between ethical leadership and employee well-being at work. Therefore, future research should explore more variables to improve the theoretical model. Third, this study explored the negative effects of ethical leadership using employee’s well-being at work as dependent variable. It cannot fully reveal the extent of the negative effects of ethical leadership. Future research should further enrich the research on the negative effects of ethical leadership by introducing alternative dependent variables, and also verify the conclusions of this study. Fourth, the meta-analysis of Hoch et al. (2018) showed that transformational leadership has a stronger explanatory power and advantages in predicting organizational citizenship behavior than ethical leadership. Therefore, it is suggested that subsequent research should analyze the influence of transformational leadership on organizational citizenship anxiety, and subsequently further expand the conclusions of this research study.

## Data Availability Statement

The datasets generated for this study are available on request to the corresponding author.

## Ethics Statement

The studies involving human participants were reviewed and approved by the Hainan University. The patients/participants provided their written informed consent to participate in this study.

## Author Contributions

All authors listed have made a substantial, direct and intellectual contribution to the work, and approved it for publication.

## Conflict of Interest

The authors declare that the research was conducted in the absence of any commercial or financial relationships that could be construed as a potential conflict of interest.
